# Isolation and Identification of a New Tetrodotoxin-Producing Bacterial Species, *Raoultella terrigena*, from Hong Kong Marine Puffer Fish *Takifugu niphobles*

**DOI:** 10.3390/md9112384

**Published:** 2011-11-14

**Authors:** Vincent Chung-Him Yu, Peter Hoi-Fu Yu, Kin-Chung Ho, Fred Wang-Fat Lee

**Affiliations:** 1 Department of Applied Biology and Chemical Technology, The Hong Kong Polytechnic University, Hong Kong, China; E-Mails: vincentyu@ymail.com (V.C.-H.Y.); bcpyu@inet.polyu.edu.hk (P.H.-F.Y.); 2 School of Science and Technology, The Open University of Hong Kong, Hong Kong, China; E-Mail: kcho@ouhk.edu.hk

**Keywords:** tetrodotoxin, TTX-producing bacteria, *Raoultella terrigena*, *Takifugu niphobles*

## Abstract

Puffer fish, *Takifugu niphobles*, collected from the Hong Kong coastal waters were screened for tetrodotoxin-producing bacteria. A Gram-negative, non-acid-fast, non-sporing and rod shaped bacterial strain (designated as gutB01) was isolated from the intestine of the puffer fish and was shown to produce tetrodotoxin (TTX). Based on the Microbial Identification (MIDI) and 16S-23S rDNA internal transcribed spacer (ITS) phylogenetic analysis, the strain was identified as *Raoultella terrigena.* The TTX production ability of the strain was confirmed by mouse bioassay, ELISA and mass spectrometry (MALDI-TOF). Our results reiterate that the TTX found in puffer fish was likely produced by the associated bacteria and TTX are widely produced amongst a diversity of bacterial species.

## 1. Introduction

Tetrodotoxin (TTX), commonly known as puffer fish toxin, is one of the most lethal neurotoxins and is also known as the causative agent of puffer fish poisoning (PFP). Puffer fish is the most recognizable living organism that contains TTX. The amount of TTX in the puffer fish are species specific and varies among different organs in different seasons [1,2]. The origin of TTX in the TTX bearing organisms has been extensively studied in the case of puffer fish. It was reported that cultivated puffer fish above the sea bed or in an enclosed water system was found to be non-toxic, but they can become toxic when they were grown in open water again or were fed with toxic puffer livers [3]. These experimental findings suggested that TTX can be acquired and accumulated from the food chain in the higher level TTX bearing organism, like puffer fish. In addition, recent studies demonstrated that, besides puffer fish, TTX is widely distributed amongst a wide range of organisms and animals. Therefore, it has been suggested that TTX has an exogenous microbial origin, rather than being produced by puffer fishes *per se* [4].

TTX-producing bacteria have been isolated from various marine organisms and therefore they have been considered as one of the possible sources for TTX production in many TTX-bearing marine organisms [5–7]. *Vibrio* was the first reported TTX-producing bacterial strain isolated from the intestine of xanthid crab [5]. Thereafter, numerous bacterial strains with TTX producing ability were isolated and identified [4]. The number and types of TTX-producing bacterial strains have been increasing and most of the reported strains were belong to the genus *Vibro*, such as *Vibrio alginolyticus* isolated from puffer fish [8,9], starfish [10] and gastropod [11]. Other than the *Vibro* species, bacterial species including *Pseudomonas* spp., *Bacillus* spp., *Aeromonas*, *Actinomyces*, *Serratia*, *Microbacterium*, were other commonly found TTX producing species [7,9,12–16]. For the past two decades, more than 10 different TTX-producing bacterial strains have been isolated and identified from various organs of puffer fish (Table 1). The bacteria were mostly isolated from the ovary, liver and intestine, since these organs have been reported to be the most toxic tissues in various TTX-bearing organisms. Although there are more than 60 indigenous species of puffer fishes reported in China Sea [9], studies conducted to investigate the TTX-producing bacteria contained in local puffer fishes are very limited [9].

The amount of TTX in puffer fish is not evenly distributed, but concentrated in several organs. In general, ovary and liver are believed to be the most toxic organs with the highest TTX level in puffer fish. However, the toxicity levels and the distribution of the toxin in different organs of puffer fish appear to be species specific. For example, it has been shown that the toxicity of the intestines of Japanese puffer fish *Takifugu niphoble* was comparable to the ovaries and livers, but far more potent than the other organs [15].

In the present study, we report a newly TTX-producing bacterial species, *Raoultella terrigena*, which was isolated from the intestines of a local toxic puffer fish *Takifugu niphoble.* The identity of the strain was investigated by MIDI and sequencing of the 16S-23S rDNA ITS region. Toxicity and TTX-producing ability of the strain were determined by mouse bioassay, ELISA and mass spectrometry. Analysis on the morphological and physiological characteristics of the strain was also performed.

## 2. Results and Discussion

### 2.1. Bacterial Strain Isolation and Determination of TTX Production

In the present study, we isolated totally five bacterial strains from the intestine of a local puffer fish *Takifugu niphobles* and assayed their toxicity. Mouse bioassay revealed that one of the strains, designated as gutB01, showed its ability to produce toxicity. Hence, further characterization of this strain was performed.

In the mouse bioassay, mice injected with the toxin extracted from gutB01 showed typical symptoms of TTX intoxication, such as convulsions and dyspnea, and were killed within 10 to 15 min. The toxicity of gutB01 determined by the mouse bioassay was 7.7 μg/L and 4.2 μg/L of cells cultivated for 24 h and 48 h respectively (Table 2). ELISA methods have been successfully demonstrated to determine the presence of TTX in bacteria isolated from the gastropod *N. semiplicatus* and ovary of puffer fish *Takifugu obscures* previously [21,24]. Here, this competitive ELISA method was used to further determine the TTX production from the strain gutB01. The standard curve established in the experiment with a *R*^2^ value 0.985 showed a good linearity relationship between absorbance signal and the TTX concentration within the range of 0–100 ng/mL (data not shown). The TTX concentration of the strain gutB01 that cultivated for 24 h was 4.3 μg/L and it was calculated by the regression equation derived from the standard curve (y = −0.049In(x) + 0.006). There was a discrepancy between the results obtained from the mouse bioassay and ELISA. The presence of some unidentified toxic components, or TTX derivatives may contribute to the higher toxicity observed in the mouse bioassay. Furthermore, the toxicity of cells cultured for 24 h is two-fold higher than that of the cells cultured for 48 h (Table 2). The differences in toxicity might be attributed to the different growth phases of the cells. As indicated from the growth curve, the cells cultured for 24 h were in log phase and the cells cultured for 48 h were in stationary phase. However, further studies are required to determine the effects of different growth phases/conditions to the toxicity of the bacterial cells.

**Figure 1:**
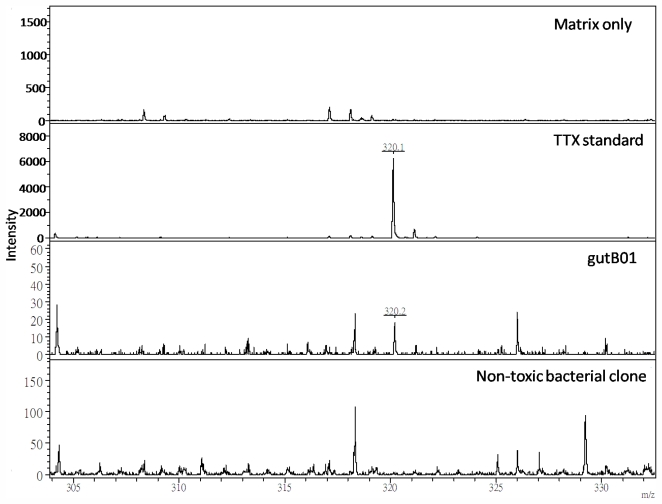
MALDI-TOF mass spectra of toxin extract isolated from gutB01. Only the TTX standard and sample of gutB01 exhibited the mass of 320.2 Da [M + H]^+^ (corresponding to the mass of protonated ion of TTX), but not the matrix and toxin extract from a non-toxic bacterial clone.

Mass spectrometry, such as ESI-MS, ESI-Q-TOF MS and MALDI-TOF MS, has been used extensively to detect the presence of TTX in suspected organisms. For example, the occurrence of TTX in the Brazilian frog *Brachycephalus ephippium* was demonstrated by MALDI-TOF MS previously [25]. The chemical identity of putative TTX purified from the isolated strain gutB01 was further analyzed by MALDI-TOF MS (Figure 1). In a positive detection mode, a distinctive peak ion at *m/z* 320.2 [M + H]^+^ was observed in both TTX standard and toxin purified from strain gutB01. Such protonated molecular ion (320.2 [M + H]^+^) was compatible to the corresponding molecular mass of TTX (319 Da). Since the corresponding peak mass ion at *m/z* 320.2 [M + H]^+^ was only observed in the standard and gutB01 toxin sample but not the non-toxic bacterial isolate and the matrix *per se*, we believe the putative TTX in the toxin sample purified from the cultured strain gutB01 could most likely be identified as authentic tetrodotoxin. Although we were not able to determine the presence of TTX analogues due to the lack of the corresponding toxin reference standards, toxicity results obtained from the three assays allowed us to believe that strain gutB01 bears TTX-producing ability.

### 2.2. Identification and Phylogenetic Analysis of Strain GutB01

Strain gutB01 was subjected to the MIDI analysis (Table 2), which has been one of the most common methods used for microbial identification. The basic principle of this method is to match the fatty acid profile of the unknown species to the fatty acid profiles of various reference microbial species in a database. The MIDI results showed that the bacterial isolate gutB01 was *Raoultella terrigena*, with high similarity index 0.906 (similarity index 1.00 indicates the exact match of the fatty acid profiles between the sample and suggested strain in the database).

**Figure 2:**
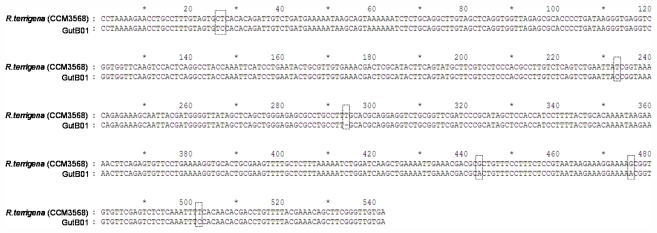
16S-23S rDNA ITS sequences of the strain gutB01. The most similar sequence obtained from bioinformatic search against the NCBInr database was the sequence from *Raoultella terrigena* (CCM3568, Accession no. EU623235). The alignment of both sequences and their differences are shown (nucleotides highlighted with dotted lines). There are 7 mismatches/gaps between these two sequences (with 542 nucleotides).

**Figure 3:**
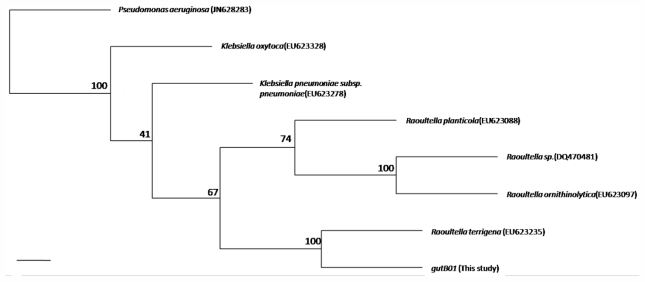
Neighbor-joining tree showing the phylogenetic relationship of the present strain gutB01 with other *Raoultella* (*Klebsiella*) species based on the ITS (16S-23S rDNA ITS) sequences. The numbers represent the percentage of 1000 replications (bootstrap support) for which the same branching patterns were obtained. *Pseudomonas aeruginosa* (JN628283) is employed as the outgroup reference. NCBI accession numbers of the ITS sequences used for the study are EU623235 (*Raoultella terrigena*), EU623088 (*Raoultella planticola*), EU623097 (*Raoultella ornithinolytica*), DQ470481 (*Raoultella* sp.), EU623278 (*Klebsiella pneumonia* subsp. *pneumoniae*), and EU623328 (*Klebsiella oxytoca*). The scale bar indicates 1% divergence.

For the past decades, phylogenetic and species identification studies based on 16S-23S rDNA ITS sequences have begun to appear more frequently in the literature and is becoming one of the most important tools for bacterial species identification [26,27]. Such a sequence was chosen in the molecular analysis of gutB01, because this region provides a more accurate representation of microbial genotypes. For example, a previous study to analyze the 16S-23S rDNA ITS sequences of *Klebsiella* species reveals that the ITS region bear sufficient variations to allow differentiation between the bacterial species [28]. The sequence obtained in our study was searched against the NCBInr database through the BLAST program. The species with the most similar sequences (99% identical) resulted from the searches was *Raoultella terrigena* (CCM3568, Accession no. EU623235). When the ITS sequence of *Raoultella terrigena* aligned with the corresponding sequence of gutB01 (Figure 2), there were only 1.3% of mismatched nucleotides or gaps found between the two sequences. Followed by the sequence analysis, we performed phylogenetic analysis based on the 16S-23S rDNA ITS sequences (Figure 3). Although the results may be affected due to the lack of other *Raoultella* (*Klebsiella*) sequences data available for the phylogenetic analysis, the tree clearly showed that the strain gutB01 fall within the cluster comprising the members of genus *Raoultella* (*Klebsiella*) and was closest to the species *Raoultella terrigena.*

### 2.3. Morphological and Physiological Characteristics of Strain GutB01

**Figure 4:**
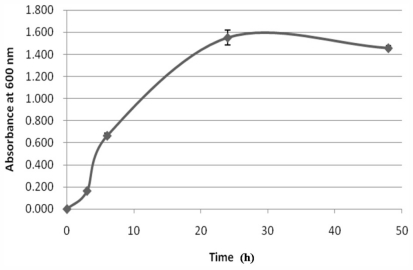
Growth curve of bacterial strain gutB01. The growth was monitored through optical density measurement of culture medium at wavelength 600 nm.

Based on the morphological and physiological analyses (Table 3), the bacterial strain gutB01 was found to be non-sporing, non acid-fast and Gram-negative rod shaped. The colonies of the strain were circular, convex, and had a diameter of between 2 and 4.5 mm. Growth was observed between 20 °C and 37 °C, 0% and 3% NaCl, pH 7 and 9. The growth curve of the strain gutB01 is shown in Figure 4. As can be seen from the curve, the strain reached the end of the log phase after 24 h incubation.

All in all, based on all these data obtained, along with the data from the MIDI analysis, sequence alignment of the 16S-23S rDNA ITS as well as phylogenetic analysis of the species presented in the study, suggests that the present toxic strain gutB01 was identified as *Raoultella terrigena*.

## 3. Experimental Section

### 3.1. Puffer Fish Samples and Collection

Specimens of puffer fish *Takifugu niphobles* were collected along the Hong Kong coastal waters in January 2006. The collected specimens were kept alive and transported to the laboratory. Various organs including the intestine of each fish were sampled aseptically for bacteriological examination and toxicity assay.

### 3.2. Bacterial Culture Medium and Agar Plates

Sterilized Ocean Research Institute (ORI) medium was used for culturing bacteria [30], which contained (in gram per liter) protease peptone No. 3 (Difco), 2 g; Bacto-yeast extract (Difco), 2 g; Phytone peptone (BBL), 1 g; sodium thiosulphate, 0.4 g; sodium sulphite, 1 g; iron citrate, 0.08 g; seawater, 750 g and adjusted to pH 7.6, with sodium hydroxide or hydrochloric acid. The ORI agar plates were prepared with 15 g of agar dissolved in 1 L of ORI medium.

### 3.3. Bacterial Isolation

Bacteria were extracted from the puffer fish intestine by adding 10 mL of Phosphate Buffered Saline (PBS) to 2 g of target organ. The tissue extract was prepared by blending the tissue for 1 min with homogenizer in ice bath. Tissue residue and fat were filtered off and subjected to serial dilutions (10^−1^ and 10^−2^) using PBS. 100 μL of each diluted sample inoculated to the ORI agars using streak plate method. The plates were maintained at 30 °C for 3–5 days to allow bacterial growth. Each discrete colony was further subcultured for several times to ensure that it was a pure culture before subjected to the toxicity test and bacterial identification.

### 3.4. Extraction and Purification of TTX

The extraction and purification of TTX from the samples were described previously [9]. Briefly, the cultures grown for 24 or 48 h were centrifuged at 8000 rpm for 30 min to remove the bacterial cells. The supernatant was collected and evaporated in reduced pressure to concentrate, which was then added to pre-washed activated charcoal under agitation and filtered through a Buchner funnel with autoclaved cheesecloth. The charcoal on the funnel was thoroughly washed with distilled water and 1% acetic acid in 20% aqueous ethanol was used to elute the adsorbed TTX. The eluate was concentrated and subjected to gel-filtration through a column of Bio-Gel^®^ P-2 (Bio-Rad Laboratories) which was equilibrated with 0.03 M acetic acid. Toxic fractions were then combined and subjected to cation exchange chromatography through a column of Bio-Rex^®^ 70 (H^+^ form, Bio-Rad Laboratories). The TTX was eluted by a linear gradient using 0–0.03 M acetic acid. Toxic fractions were combined and concentrated for further toxicity bioassay and detection of TTX.

### 3.5. Assays for TTX

#### 3.5.1. Mouse Bioassay

Mouse bioassay was performed as described [9]. Briefly, healthy mice (Institute of Cancer Research, ICR) which weighed between 15–25 g were used in the mouse bioassay. Toxin samples used in the mouse bioassay are purified by 0.2 μm syringe filter before the injection. A group of 4–6 mice were chosen randomly and 1 mL of toxin sample was injected intraperitoneally into the mice. The death time was recorded at the last grasping breath of the mouse. Lethal potency of TTX was expressed in mouse units (MU), one MU was defined as the amount of toxin required to kill a 20 g ICR strain mouse in 30 min after intraperitoneal injection and specific toxicity of pure TTX is (5000 MU/mg).

#### 3.5.2. Enzyme-Linked Immunosorbent Assay (ELISA)

The ELISA kit used in the present study was kindly provided by The Institute of Food Quality and Safety, South China Agriculture University, Guangzhou [31]. For the preparation of the ELISA plate, 2 μg/mL TTX-OVA coating antigen was coated to microplate with coating solution for about 18 h and the residual liquid was tapped out. 200 μL blocking solution (5% skimmed milk powder in PBS) was then used to mask the uncoated region and prevent the non-specific binding. It was followed by washing with washing buffer PBST (PBS with 0.05% Tween-20) for 5 times after 2 h incubation. To detect TTX concentration in the samples, 50 μL diluted peroxidase conjugated antibody (diluted with PBST with 1% BSA) into each well following by adding 50 μL TTX standard or sample into the wells (diluted with PBST if needed). The microplate was incubated for 30 min at room temperature. Washing step was repeated and 100 μL TMB (3,3′,5,5′-Tetramethylbenzidine) substrate solution was added into each well and incubated at 37 °C for 15 min. 50 μL stopping solution (2 M sulfuric acid) was then added into each well to stop the reaction. The absorbance was measured at wavelength 450 nm and the well with PBST was used as blank. Standard curve was constructed with standard TTX at the concentration range of 0–100 ng/mL. TTX concentration of the sample was calculated based on the standard curve.

#### 3.5.3. Mass Spectrometry

The toxin samples were analyzed by MALDI-TOF mass spectrometry (Autoflex III, Bruker, Germany). 1 μL of sample solution were mixed with 1 μL of matrix solution. The resulting mixtures were then vortexed before spotting 0.5 μL volume onto a mass spectrometer target plate (MTP AnchorChip™ 600/384) (Bruker, Germany). Sample mixtures were crystallized at room temperature, and their masses analyzed in reflectron mode at an accelerating voltage of 20 kV by using a 100 ns delay time over a mass range of 100–1000 Da using external mass calibration with calibration standards from the manufacturer. Matrix alone and a non-toxic bacterial clone isolated from the same puffer fish were used as control.

### 3.6. Identification of TTX Producing Bacteria

#### 3.6.1. MIDI Identification System

Isolated bacteria were characterized using The Sherlock^®^ Microbial Identification System (MIS) (MIDI, Inc., Newark, Delaware, USA), which identifies bacteria by comparison of the whole cell fatty acid profiles between the samples and the system’s database, using gas chromatography analysis.

#### 3.6.2. 16S-23S ITS DNA Sequencing and Phylogenetic Analysis

Genomic DNA of the isolated bacteria was extracted by a DNA extraction kit (Roche, Switzerland) following mechanical cell disruption by a quick homogenization. 16S-23S rDNA ITS region was amplified from the extracted DNA using PCR with the primers Raou-ITSF: 5′-CCTRAAAGAACCTGCCTTTGTAG-3′ and Raou-ITSR: 5′-TCACAACCCGAARCTGTTTCGTA-3′. PCR were performed under conditions: 95 °C 5 min; 35 cycles of 94 °C 45 s, 55 °C 45 s and 72 °C 2 min; 72 °C 10 min. PCR products were cloned into pGEM-T easy vectors (Promega, USA) prior to DNA sequencing. DNA sequencing of all cloned plasmids were performed by commercial facilities using traditional dideoxy-methodolgy. The 16S-23S rDNA ITS sequence was compared with sequences in the NCBI GenBank database using the BLAST program.

For phylogenetic analysis, the ITS sequence of gutB01 was aligned and compared with the corresponding sequences of other *Raoultella* species available from PubMed by using the Clustal X program [32]. Phylogenetic analysis was carried out using PHYLIP, version 3.69 (Joe Felsenstein, Department of Genetics, University of Washington). Distance matrices were produced using the DNADIST module according to the two-parameter model of kimura [33] and building a Neighbor-Join tree with the NEIGHBOR module. One thousand bootstrap replicates were generated and performed using the SEQBOOT module and the consensus tree was generated using CONSENSE. Tree was viewed with the Treeview software [34].

## 4. Conclusions

The present study revealed for the first time that a novel TTX-producing bacteria species, *Raoultella terrigena*, was isolated from the toxic puffer fish *Takifugu niphobles* collected from Hong Kong coastal waters. This further suggested that the toxin found in puffer fish was likely produced by the associated bacteria. However, more research is still required to elucidate the mechanism of the synthesis of TTX by bacteria, as well as their role in toxin production. In addition, our study could not exclude the possibility that other bacteria in the puffer fish *T. niphobles* may also produce TTX.

## Figures and Tables

**Table 1:** Tetrodotoxin (TTX)-producing bacteria isolated from puffer fish from 1987–2011.

Year	TTX-Producing Bacteria	Origin of Isolation	References
1987	*Pseudomonas* species	Skin of *Takifugu poecilonotus*	[7]
1987	*Vibrio alginolyticus*	Intestine of *Takifugu vermicularis*	[8]
1989	*Shewanella putrefaciens*	Intestine of *Takifugu niphobles*	[16]
2000	*Vibrio* species	Intestine of *Takifugu vermicularis radiates*	[17]
2004	*Microbacterium arabinogalactanolyticum*	Ovary of *Takifugu niphobles*	[9]
2004	*Serratia marcescens*	Skin of *Chelonodon patoca*	[9]
2004	*Vibrio alginolyticus*	Intestine of *Takifugu alboplumbeus*	[9]
2005	*Actinomyces* species	Ovary of *Fugu rubripes*	[18]
2005	*Bacillus* species	Ovary, liver, and Intestine of *Fugu rubripes*	[18]
2005	*Nocardiopsis dassonillei*	Ovary of *Fugu rubripes*	[19]
2007	*Proteobacteria*, CFB group, and *Spirochaetales*	Skin, intestine, ovary and liver of *Takifugu obscures*	[20]
2010	*Aeromonas* species	Ovary of *Takifugu obscures*	[21]
2010	*Bacillus* species	Ovary of *Fugu obscures*	[22]
2010	*Lysinibacillus fusiformis*	Liver of *Fugu obscures*	[23]
2011	*Raoultella terrigena*	Intestine of *Takifugu niphobles*	(Present study)

**Table 2:** Toxicity and the MIDI result of gutB01.

	*Raoultella terrigena* (gutB01)
Culture medium (time for cultivation)	ORI (24 h)
Mouse bioassy (MU/L)	38.5
Mouse bioassy (μg/L)	7.7
ELISA (μg/L)	4.3
Culture medium (time for cultivation)	ORI (48 h)
Mouse bioassy (MU/L)	21.1
Mouse bioassy (μg/L)	4.2
ELISA (μg/L)	N.A +

MIDI Similarity index #	0.906

# According to The Sherlock^®^ Microbial Identification System (MIDI System) manual, strains with a similarity of 0.600 or higher is considered good library comparisons. If the similarity index is between 0.400 and 0.600, it may be a good match but an atypical strain. Values lower than 0.400 suggest that the system does not have the species in the database, but indicate the most closely related species.

+ Data not available.

**Table 3:** Morphological and physiological characteristics of *Raoultella terrigena*.

Characteristic #	Raoultella Terrigena* (gutB01)
Colony diameter (mm)	2–4.5
Shape of colonies	Circular
Elevation of colony	Convex
Colony margin	Entire
Cell shape	Rod
Cell width (μm)	0.5–0.8
Cell length (μm)	0.5–0.8
Gram stain	−
Acid-fast stain	−
Spore Formation	−
Growth at	
20 °C	+
25 °C	+
30 °C	+
37 °C	+
Growth in NaCl of	
0%	+
3%	+ (little)
5%	− (decrease steadily)
7%	− (decrease steadily)
10%	− (none)
Growth at pH	
5	−
6	−
7	+
8	+
9	+

# The characteristics were measured as described previously [9].

* Other typical phenotypic characteristics of *Raoultella terrigena* are described in [29]. +: positive; −: negative.
